# Supplementary material for: TRPV4 Mediates Alveolar Epithelial Barrier Integrity and Induces ADAM10-Driven E-Cadherin Shedding
Source: Cells. 2024 Oct 17;13(20):1717. doi: 10.3390/cells13201717 (PMC11506556; doi:10.3390/cells13201717)
Supplement: Supplementary file 1 [file cells-13-01717-s001.zip › cells-3248583 WB (S2B C) (S5D-F).pdf]

Original Images for Blots

TRPV4 mediates alveolar epithelial barrier integrity and induces ADAM10-driven E-cadherin shedding

Lena Schaller, Thomas Gudermann and Alexander Dietrich

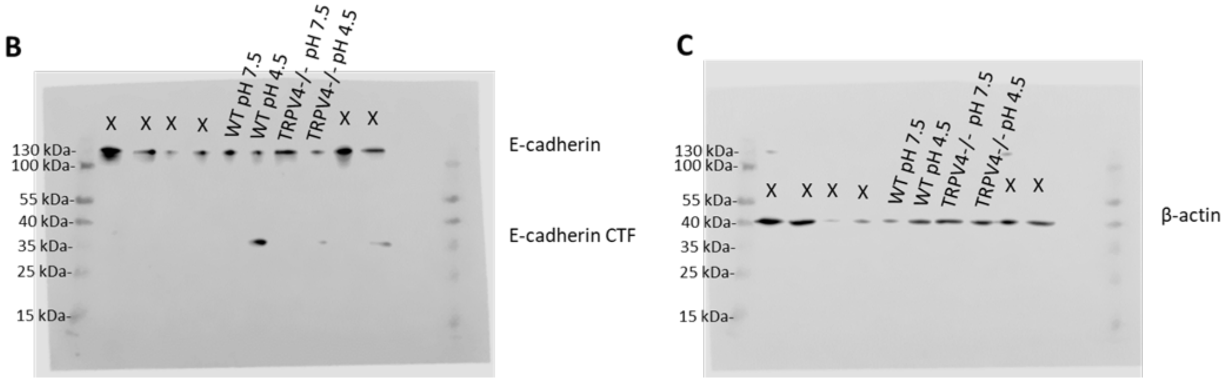

Original Blots for Figure 2C: Full scan of representative Western blot of pH-induced E-cadherin cleavage 1 h after a change in media pH. Images depict bands for E-cadherin and E-cadherin CTF (B), as well as for the loading control, β-actin (C).

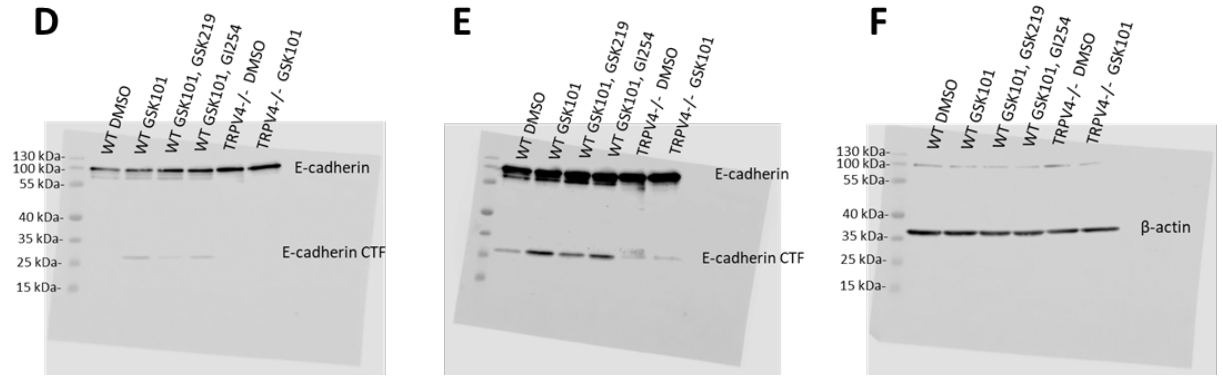

Original Blots for Figure 4A. Full scan of representative Western blot of pH-induced E-cadherin cleavage 1 h after a change in media pH. Images depict bands for E-cadherin and E-cadherin CTF at optimal brightness/contrast for the respective bands (D, E), as well as the loading control, β-actin (F).
