# Supplementary material for: TRPV4 Mediates Alveolar Epithelial Barrier Integrity and Induces ADAM10-Driven E-Cadherin Shedding
Source: Cells. 2024 Oct 17;13(20):1717. doi: 10.3390/cells13201717 (PMC11506556; doi:10.3390/cells13201717)
Supplement: Supplementary file 1 [file cells-13-01717-s001.zip › cells-3248583-supplementary S2A.pdf]

TRPV4 mediates alveolar epithelial barrier integrity and induces ADAM10-driven E-cadherin shedding

Lena Schaller, Thomas Gudermann and Alexander Dietrich

Supplementary Information

Table S1: Antibodies used for cell isolation, Western blotting and immunocytochemistry

Primary antibodies	Supplier	Cat. # / RRID	Dilution
E-Cadherin (mo pAb)	BD Biosciences	610181 / AB_397580	WB: 1:1000
B-actin-HRP (mo pAb)	Merck	A3854 / AB_262011	WB: 1:10000
Aquaporin 5 (AQP5) (rb pAb)	Alomone Labs	AQP005 / AB_2039736	ICC: 1:200
Prosurfactant protein C (pSPC) (rb pAb)	Merck	AB3786 / AB_91588	ICC: 1:200
CD16/CD32	BD Pharmingen	553142 / AB_394656	Iso: 1:666
CD45	BD Pharmingen	553076 / AB_394606	Iso: 1:666

Secondary antibodies	Supplier	Cat. # / RRID	Dilution
Mouse-HRP	Cell Signaling	7076 / AB_330924	WB: 1:10000
Rabbit Alexa Fluor 488	ThermoFisher Scientific	A32731 / AB_2633280	ICC: 1:250

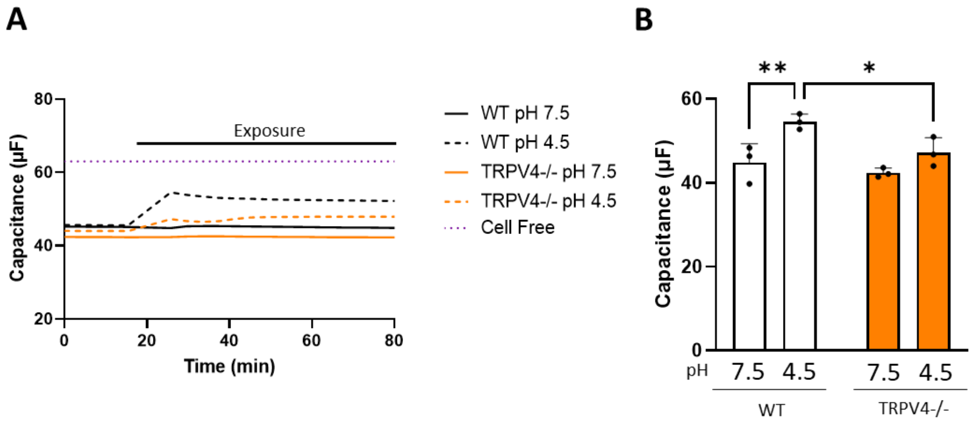

Figure S1: Low pH induces TRPV4-mediated changes in cell adhesion. (A) Capacitance values for WT and TRPV4-/- AT1 cells upon an HCl-induced change in media pH were recorded using an ECIS system at 500 Hz. Capacitance values 10 minutes after media change (pH 7.5 or pH 4.5) were quantified (B). Data represent the mean ± SD (B) of 3 independent cell preparations from 6 mice each. Significance was assessed using a two-way ANOVA. * $p < 0.05$, ** $p < 0.01$

A

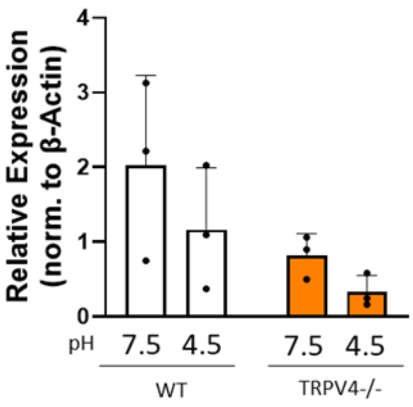

Figure S2: Low pH does not significantly change E-cadherin protein expression in AT1 cells. Quantification of Western blot results for E-cadherin protein expression in AT1 cells 1 h after a change in media pH. Data represent the mean ± SD of 3 independent cell preparations from 6 mice each. Significance was assessed using a two-way ANOVA.

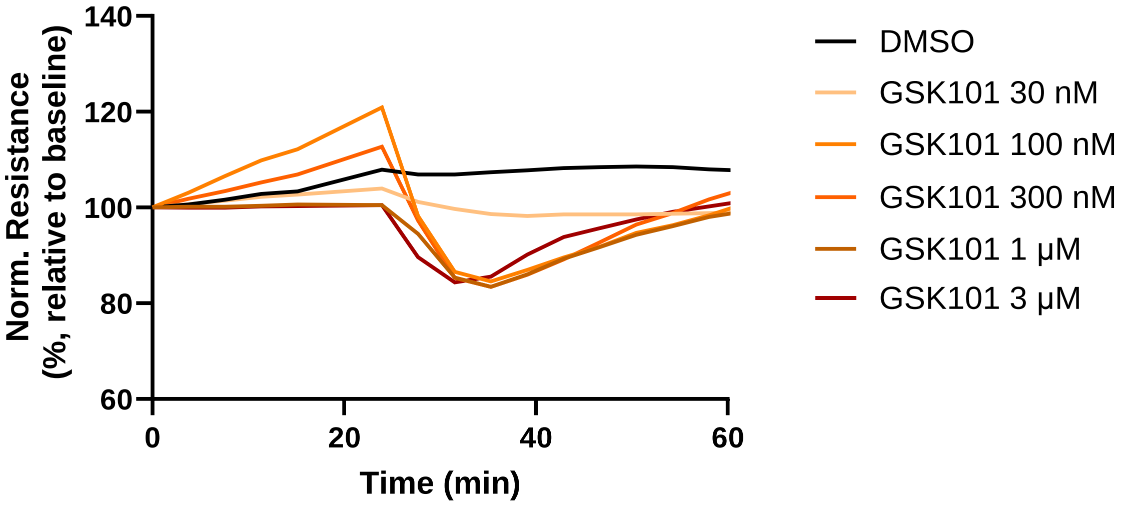

Figure S3: Loss of AT1 cell resistance upon GSK101 application plateaus at 100 nM concentration. Changes in normalized electrical cell resistance were recorded at 500 Hz in WT AT1 cells upon application of increasing concentrations of GSK101. Data represent the results from one cell preparation of 5 mice.

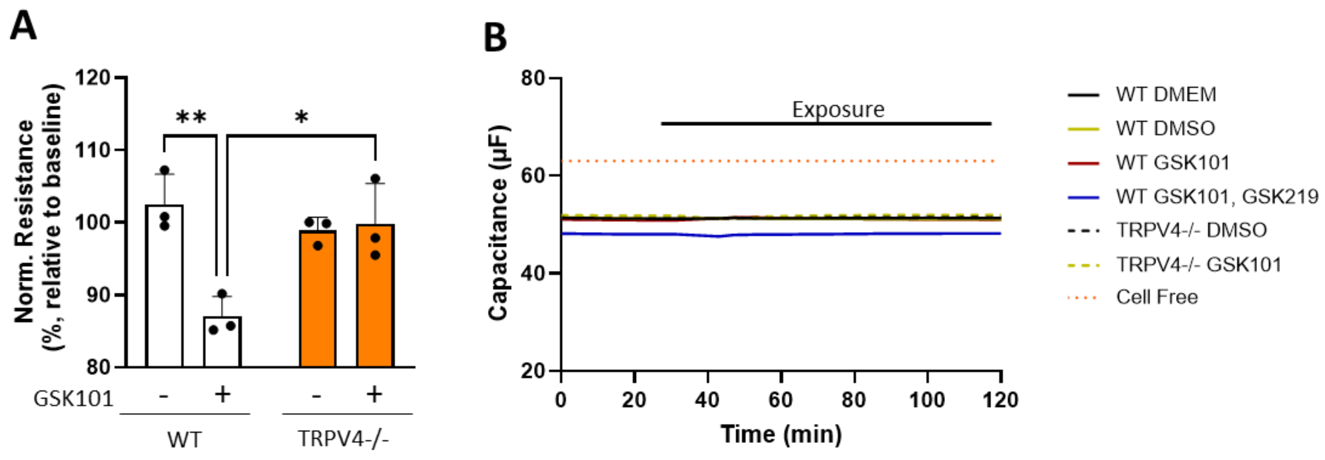

Figure S4: GSK101-induced loss of AT1 barrier resistance is TRPV4-dependant, and does not cause cell detachment. (A) The normalized electrical cell resistance for WT and *Trpv4*-/- AT1 cells 15 minutes after GSK101 (100 nM) exposure was quantified. (B) Mean capacitance values for WT and *Trpv4*-/- AT1 cells upon application of GSK101 (100 nM) in the presence and absence of GSK219 (300 nM) were recorded using an ECIS system at 500 Hz. Data represent mean ± SD (A) from 3 independent cell isolations from 5 mice each. Significance between means was analyzed with a two-way ANOVA; * $p < 0.05$, ** $p < 0.01$.

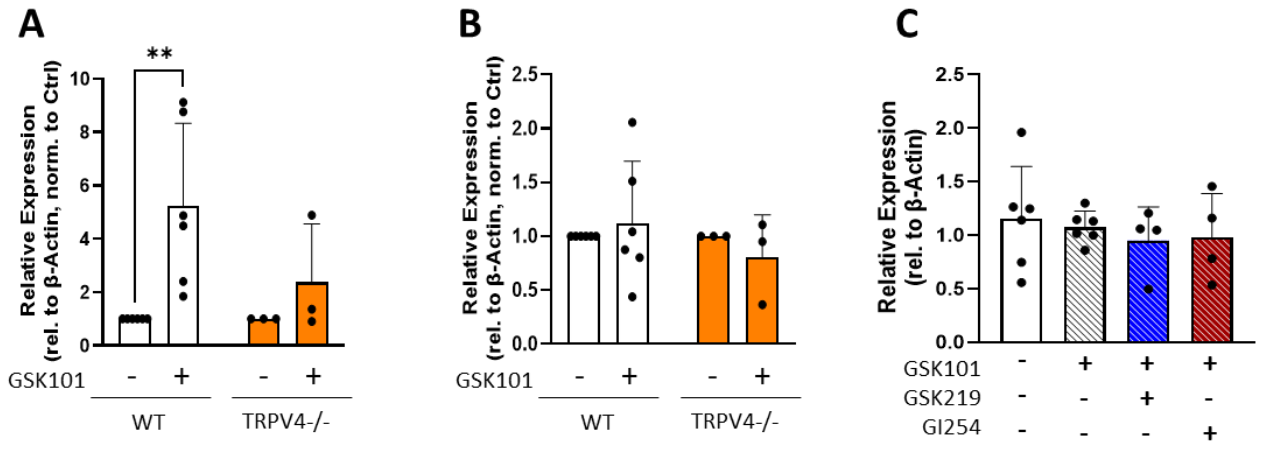

Figure S5: GSK101 exposure triggers TRPV4-dependent formation of an E-cadherin CTF, but does not significantly change the protein expression of E-cadherin. Quantification of Western blot results for the level of E-cadherin CTF (A) and E-cadherin (B) in WT and Trpv4-/- AT1 cells 15 minutes after the addition of GSK101 (100 nM). (C) Quantification of Western blot results for E-cadherin protein expression levels in WT AT1 cells 15 minutes following the addition of GSK101 (100 nM) in theh presence and absence of either the TRPV4 inhibitor GSK219 (300 nM) or the ADAM10 inhibitor GI254 (3 μM). Data represent the mean ± SD from at least 3 independent cell preparations from 3-5 mice, each. Significance was assessed using two-way (A, B) and one-way (C) ANOVA; ** $p < 0.01$.
